# Altmetric Versus Bibliometric Perspective Regarding Publication Impact and Force

**DOI:** 10.1007/s00268-018-4579-9

**Published:** 2018-03-13

**Authors:** Arfon G. M. T. Powell, Victoria Bevan, Chris Brown, Wyn G. Lewis

**Affiliations:** 1 Division of Cancer and Genetics, University Hospital of Wales, Cardiff University, Heath Park, Cardiff, UK; 2 Wales Deanery School of Surgery, Cardiff University, Heath Park, Cardiff, UK

## Abstract

**Background:**

Bibliometric and Altmetric analyses highlight key publications, which have been considered to be the most influential in their field. The hypothesis was that highly cited articles would correlate positively with levels of evidence and Altmetric scores (AS) and rank.

**Methods:**

Surgery as a search term was entered into Thomson Reuter’s Web of Science database to identify all English-language full articles. The 100 most cited articles were analysed by topic, journal, author, year, institution, and AS.

**Results:**

By bibliometric criteria, eligible articles numbered 286,122 and the median (range) citation number was 574 (446–5746). The most cited article (Dindo et al.) classified surgical complications by severity score (5746 citations). Annals of Surgery published most articles and received most citations (26,457). The country and year with most publications were the USA (*n* = 50) and 1999 (*n* = 11). By Altmetric criteria, the article with the highest AS was by Bigelow et al. (AS = 53, hypothermia’s role in cardiac surgery); Annals of Surgery published most articles, and the country and year with most publications were USA (*n* = 4) and 2007 (*n* = 3). Level-1-evidence articles numbered 13, but no correlation was found between evidence level and citation number (SCC 0.094, *p* = 0.352) or AS (SCC = 0.149, *p* = 0.244). Median AS was 0 (0–53), and in articles published after the year 2000, AS was associated with citation number (*r* = 0.461, *p* = 0.001) and citation rate index (*r* = 0.455, *p* = 0.002). AS was not associated with journal impact factor (*r* = 0.160, *p* = 0.118).

**Conclusion:**

Bibliometric and Altmetric analyses provide important but different perspectives regarding article impact, which are unrelated to evidence level.

**Electronic supplementary material:**

The online version of this article (10.1007/s00268-018-4579-9) contains supplementary material, which is available to authorized users.

## Introduction

Any reasonable observer might assume that the 100 most cited articles in surgery represent classic, landmark, or marquee communications. Certainly, the number of times articles are cited is used widely to measure the impact of journals and assess the quality of authors’ contributions. Yet it has been reported that among such best seller lists exist articles relating to topics that were “hot” or popular at one time and then faded. As time passes, even true classics are arguably cited less frequently, because their substance has been absorbed into the received wisdom of the literature—a phenomenon described as obliteration by incorporation.

A reference is the acknowledgment that one article gives to another; a citation is the acknowledgement that one article receives from another. Citation analysis is that area of bibliometrics that deals with the study of these relations. Citation analysis involves ranking and evaluating an article or journal based on the number of citations it receives. The establishment of a citation rank list identifies published work that has had the greatest intellectual influence. In addition to determining the most frequently cited articles, this analysis is also used to rank journals in terms of impact [1]. Many medical specialties have utilized citation rank analysis to identify the most influential papers in their field which include trauma and orthopaedic surgery [2], general surgery [3, 4], emergency general surgery [5], and oncology [6].

Citations take time to accumulate, and other, faster assessment means have emerged recently which has led to the development of alternative metrics, or “Altmetrics”. These extend the concept of citation beyond references in other scientific papers; by recording, for example, how often a paper is downloaded, or when the outcome of a clinical trial is used to develop guidelines for doctors, or if a piece of work is included in a course curriculum. To date, no study has undertaken to determine the most influential articles in the global field of surgery and to compare the relative value of citation number, with level of evidence or Altmetric score (AS). The aim of this study, therefore, was to determine the areas of translational surgical research that have been most influential in driving advances in the art and science of surgery, not only to identify what makes a surgical article citable, but also to develop what might arguably constitute an all-time surgical must-do reading list. The hypotheses were: firstly, higher citation number and rank would correlate positively with higher levels of evidence than lower citation number and rank; secondly, AS and rank would correlate with citation number and rank, after a certain critical publication date, given that organisations such as “twitter” have only been in existence since 2006.

## Methods

A search of the Thomson Reuters Web of Science citation-indexing database and research platform was completed using the search term Surgery. The returned dataset was filtered to include only English-language and full articles and sorted by number of citations; a method initially developed by Paladugu et al. [3]. The 100 most cited articles were identified from the large number of manuscripts returned. The dataset was then further evaluated examining title, first and senior author, institution and department of the first author, topic, specialty, year of publication, and the country of origin. The 5-year impact factor (for the year 2015) of each journal publishing the articles was recorded. The quality of evidence contained within the articles was assessed according to the Sackett scoring system [7] and the Oxford Evidence Based Medicine scoring system [8]. Altmetric scores were obtained by downloading the “Altmetric it” function from the Altmetric.com website (https://www.altmetric.com/products/free-tools/bookmarklet/) and analysed by utilising the journal article page containing the doi reference number.

## Results

The Web of Science search returned 286,122 full-length, English-language articles. Table 1 lists the 100 most cited articles [9–108]. The number of citations ranged from 5746 for Dindo et al. [9] to 446 for Kennedy et al. [108]. The median citation was 574 [interquartile range (IQR) 354.8–792.3], which was not normally distributed. The oldest article featured in the top 100 was by Bigelow et al. [69]. The most recent article was by Clavien et al. [13].

**Table 1:** The top 100 cited papers in surgery

Rank	Citations	First author	Rank	Citations	First author
1	5746	D Dindo	51	571	JD Cooper
2	1756	Y Fong	52	567	TP Grantcharov
3	1704	SAM Nashef	53	567	PF Sharkey
4	1575	RB Rutherford	54	560	HG Willert
5	1437	PA Clavien	55	560	K Maruyama
6	1242	NF Kassell	56	556	YM Fong
7	1220	RJ Heald	57	549	DA Luce
8	1168	L Norgren	58	541	O Dworak
9	1130	NE Seymour	59	538	WG Bigelow
10	1089	A Carpentier	60	535	TH Rockwood
11	1063	M Lacroix	61	527	KJ Jenkins
12	992	DN Krag	62	526	JR Steadman
13	926	JA Martin	63	524	TM Pawlick
14	853	F Rogues	64	517	R Reznick
15	833	SF Khuri	65	516	DL Morton
16	811	G Gugliemi	66	516	LL Creswell
17	800	R Earlam	67	511	WE Enker
18	784	LV Laitinen	68	510	J Marescaux
19	769	SA Curley	69	505	SS Burkhart
20	754	PR Schauer	70	504	H Kehlet
21	753	JM Porter	71	502	JC Cheville
22	751	RJ Heald	72	500	P Borgstein
23	740	R Adam	73	499	I Ciric
24	733	JR Siewert	74	491	AC Wittgrove
25	711	L Norgren	75	491	PM Black
26	697	LG Svensson	76	488	KCMJ Peeters
27	690	LM Galatz	77	488	D Rattner
28	687	JH Klinkenbijl	78	486	J Bernier
29	686	VW Fazio	79	486	LH Edmunds
30	679	B Eklof	80	484	K Slim
31	674	NV Christou	81	484	BW Lytle
32	668	ED Arrington	82	484	KC Conlon
33	665	AL Benabid	83	483	T Kajitani
34	659	G Knutsen	84	480	RMH Roumen
35	657	SM Strasberg	85	475	JH Balcom
36	646	F Vinuela	86	472	A Habr-Gama
37	645	SA Rosenberg	87	467	RM Rosenfeld
38	631	H Bismuth	88	466	JA Sosa
39	622	PA Clavien	89	465	JM Becker
40	620	B Brandstrup	90	461	C Gerber
41	618	O Ethgen	91	460	BE Bierbaum
42	614	MN Wente	92	458	CW le Roux
43	613	GP Buzby	93	458	DJ Gouma
44	605	PR Schauer	94	457	P Boileau
45	604	NF Kassell	95	452	CGS Huscher
46	600	H Kehlet	96	449	CK Zarins
47	595	J Butler	97	448	EH Oldfield
48	593	MS Chen	98	446	L Hangody
49	581	AP Furnary	99	446	M Minagawa
50	576	NT Nguyen	100	446	DW Kennedy

The 100 most cited articles were published in 30 journals with the number of articles per journal ranging from 1 to 32 (Table 2). Annals of Surgery not only published the most articles (*n* = 32), but also received the most citations (26,457). Annals of Surgery had the highest impact factor of 8.6, with a 5-year impact factor of 8.7.

**Table 2:** Journals with the top 100 cited surgery articles

Journal title	5-year impact factor	Number of articles in the top 100	Number of citations
Annals of Surgery	8.7	32	26,457
Journal of Neurosurgery	3.5	8	6263
Journal of Bone and Joint Surgery	5.4	8	4351
Journal of Vascular Surgery	3.3	6	5321
British Journal of Surgery	5.8	4	3513
Journal of Thoracic and Cardiovascular Surgery	3.5	4	2671
Annals of Thoracic Surgery	3.3	4	2178
Journal of the American College of Surgeons	5.1	4	2133
Archives of Surgery	4.9	3	1736
American Journal of Surgery	2.6	3	1730
European Journal of Cardiothoracic Surgery	2.7	2	2557
Surgery	3.7	2	1236
Clinical Orthopaedics and Related Research	3.5	2	1235
Arthroscopy—The Journal of Arthroscopic and Related Surgery	3.9	2	1031
Neurosurgery	3.3	2	990
Surgical Oncology	3.2	1	992
European Journal of Vascular and Endovascular Surgery	3.0	1	711
World Journal of Surgery	2.8	1	560
Journal of Cataract and Refractive Surgery	3.1	1	549
International Journal of Colorectal Disease	2.4	1	541
Diseases of the Colon and Rectum	3.8	1	535
American Journal of Surgical Pathology	5.1	1	502
Obesity Surgery	3.4	1	491
Surgical Endoscopy and Other Interventional Techniques	3.5	1	488
Head and Neck—Journal for the Sciences and Specialties of the Head and Neck	2.8	1	486
ANZ Journal of Surgery	1.3	1	484
Japanese Journal of Surgery	N/A	1	483
Otolaryngology—Head and Neck Surgery	2.1	1	467
Archives of Otolaryngology—Head and Neck Surgery	2.3	1	446

*N/A* not available

The country and year with the most articles in the top 100 was the USA with 50 and 1999 (*n* = 11), followed by France with 8 and 2004 (*n* = 9). The institution with the most citations was the University of Zurich with 7644 across 3 articles (supplementary Table 1). The Memorial Sloan–Kettering Cancer Center and Washington University had the highest number of articles in the top 100 with 4 (supplementary Table 1). Seven authors had 2 first author articles in the top 100 with the highest citation index of 2312.

The commonest related specialty to feature in the top 100 was hepato-pancreatico-biliary surgery with 15 articles. This was followed by Trauma & Orthopaedic surgery (*n* = 13), and Cardiothoracic surgery (*n* = 11), respectively (supplementary Table 2). The specialties with the fewest articles in the top 100 were Ophthalmology, Plastic surgery, and Urology, with one each. The most common topic to feature in the top 100 was management of surgical disease with 53 articles, which included 14 (26%) randomised control trials (supplementary Table 3). The second commonest topic was the identification, classification and management of surgical complications with 16 articles.

Of the 14 randomised control trials, 8 related to the management of cancer, 2 related to the management of aneurysmal disease, 2 related to the use of laparoscopic bariatric procedures, 1 article related to the management of post-operative adhesions, and 1 compared autologous chondrocyte implantation with arthroscopic microfracture in patients with full-thickness traumatic defects of the knee. Of those articles related to cancer, colorectal cancer (*n* = 12) was the most prevalent followed by gastric cancer (*n* = 4). The median citation of the clinical trials was 504 (IQR 351.0–656.0) with a range of 687 to 446 compared with 588.0 (IQR 336.3–839.8) with a range of 5746 to 446, *p* = 0.031 for non clinical trials (supplementary figure 1).

**Fig. 1:**
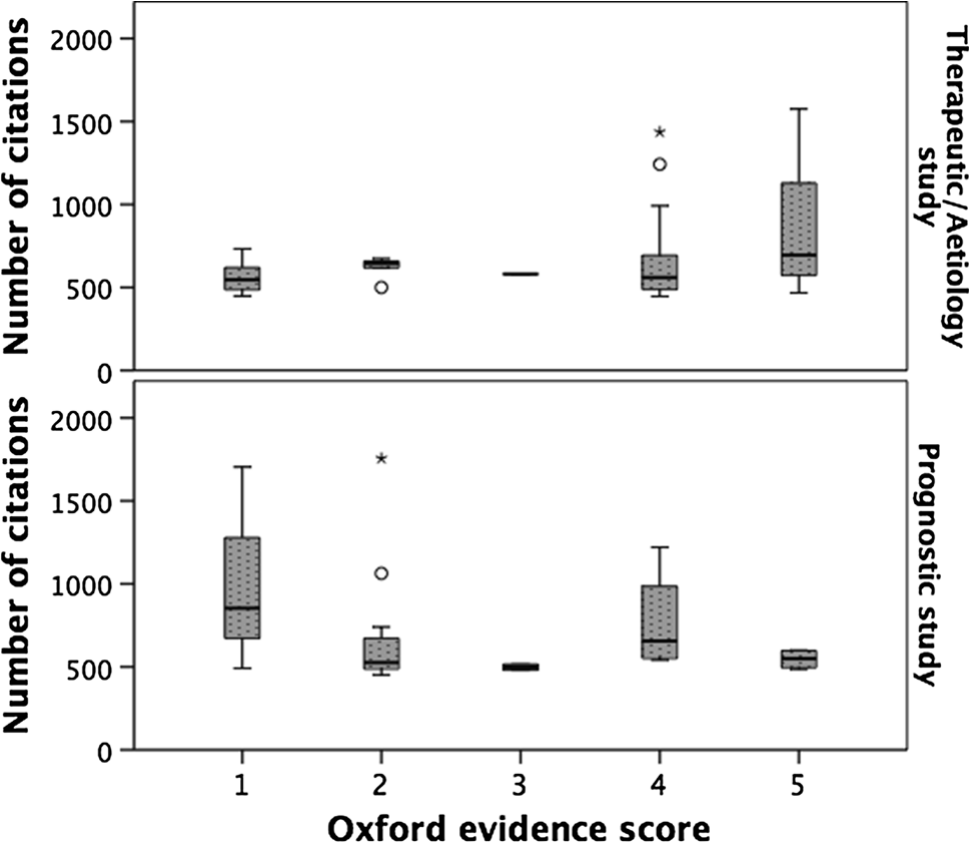
The relationship between evidence level and number of citations in manuscripts stratified by study type. *Therapeutic/aetiology studies *p* = 0.444 Kruskal–Wallis test. Prognostic studies *p* = 0.393 Kruskal–Wallis test

Evidence levels of the included articles were initially scored using the Sackett scoring method [7]. Three were level 1 evidence, 12 were level 2 evidence, 18 were level 3 evidence, 25 were level 4 evidence, 28 were level 5 evidence, and 14 were not scored as they were guidelines or consensus statements. There was no relationship between the quality of evidence and the median number of citations received (*p* = 0.674, supplementary figure 2). The median number of citations received for each evidence level was: level 1 studies 488.00 (range 486.00–516.00), level 2 584.50 (IQR 449.00–766.25), level 3 604.50 (IQR 458.00–879.25), level 4 556.00 (IQR 446.00–798.00) and level 5 554.50 (IQR 446–919.50) (supplementary figure 2). A possible limitation of the Sackett scoring system is that a well-designed prognostic study is still a cohort study that is considered low-level evidence. Therefore, the studies were also scored using the Oxford Evidence Based Medicine scoring system [8]. Studies were grouped as either therapeutic/aetiology (*n* = 69) or prognostic (*n* = 25). With regard to therapeutic/aetiology studies, there was no difference in the median number of citations received for each evidence level. The median for level 1 (*n* = 10) was 546.00, level 2 (*n* = 8) was 559.00, level 3 (*n* = 1) was 587.00, level 4 (*n* = 40) was 587.50, and level 5 (*n* = 10) was 668.00 (*p* = 0.444) (Fig. 1). For prognostic studies, there was a difference in the median number of citations received for each evidence level. The median for level 1 (*n* = 3) was 853.00, level 2 (*n* = 12) was 525.00, level 3 (*n* = 2) was 498.00, level 4 (*n* = 4) was 655.50, and level 5 (*n* = 4) was 549.50 (*p* = 0.393) (Fig. 1).

To test for a correlation between citations and evidence levels, the number of citations variable was grouped into deciles to meet the assumptions of the Spearman’s correlation coefficient (SCC) test. There was no significant correlation between number of citations and the Oxford evidence level (SCC 0.094, *p* = 0.352). When therapeutic/aetiology studies were analysed, there was a weak statistical association between low evidence level and higher citation number (SCC 0.233, *p* = 0.054); however, this was not evident for prognostic studies (RCC − 0.012, *p* = 0.955).

A possible limitation of this type of study is that historical articles may accrue a larger number of citations despite lacking the impact of newer articles. To control for this, the number of citations were divided by the number of years since publication to give a citation rate index (CRI) (Table 3) [9–13, 15, 16, 19, 33, 50]. The CRI for the top 10 articles ranged from 442.00 for Dindo et al. [9] to 61.40 for Wente et al. [50]. The USA had the most articles in the top 10 CRI with 4, followed by Sweden and Switzerland with 2 each. Management of complications (*n* = 3) and consensus statements on the management of peripheral vascular disease (*n* = 3) were the commonest topics in the top 10 CRI. The citation rate analysis for clinical trials ranged from 53.91 to 18.43 compared with 442.00 to 8.03 for all 100 articles studied.

**Table 3:** Top 10 articles with the highest citation rate

Rank	Citation rate	First author	Senior author	Title	Institution	Country
1	442.00	D. Dindo	PA. Clavien	Classification of surgical complications—A new proposal with evaluation in a cohort of 6336 patients and results of a survey	University of Zurich	Switzerland
2	179.63	PA. Clavien	M. Makuuchi	The Clavien-Dindo Classification of Surgical Complications Five-Year Experience	University of Zurich	Switzerland
3	116.80	L. Norgren	CD. Liapis	Inter-society consensus for the management of peripheral arterial disease (TASC II)	Orebro University	Sweden
4	97.56	Y. Fong	LH. Blumgart	Clinical score for predicting recurrence after hepatic resection for metastatic colorectal cancer—Analysis of 1001 consecutive cases	Memorial Sloan–Kettering Cancer Center	USA
5	94.67	SAM. Nashef	R. Salamon	European system for cardiac operative risk evaluation (EuroSCORE)	Papworth Hospital	England
6	78.75	RB. Rutherford	DN. Jones	Recommended standards for reports dealing with lower extremity ischemia: Revised version	University of Colorado	USA
7	75.33	NE. Seymour	RM. Satava	Virtual reality training improves operating room performance—Results of a randomized, double-blinded study	Yale University School of Medicine	USA
8	71.10	L. Norgren	CD. Liapis	Inter-society consensus for the management of peripheral arterial disease (TASC II)	Orebro University	Sweden
9	66.44	M. Lacroix	R. Sawaya	A multivariate analysis of 416 patients with glioblastoma multiforme: prognosis, extent of resection, and survival	The University of Texas	USA
10	61.40	MN. Wente	MW. Buechler	Delayed gastric emptying (DGE) after pancreatic surgery: a suggested definition by the International Study Group of Pancreatic Surgery (ISGPS)	University of Heidelberg	Germany

**Fig. 2:**
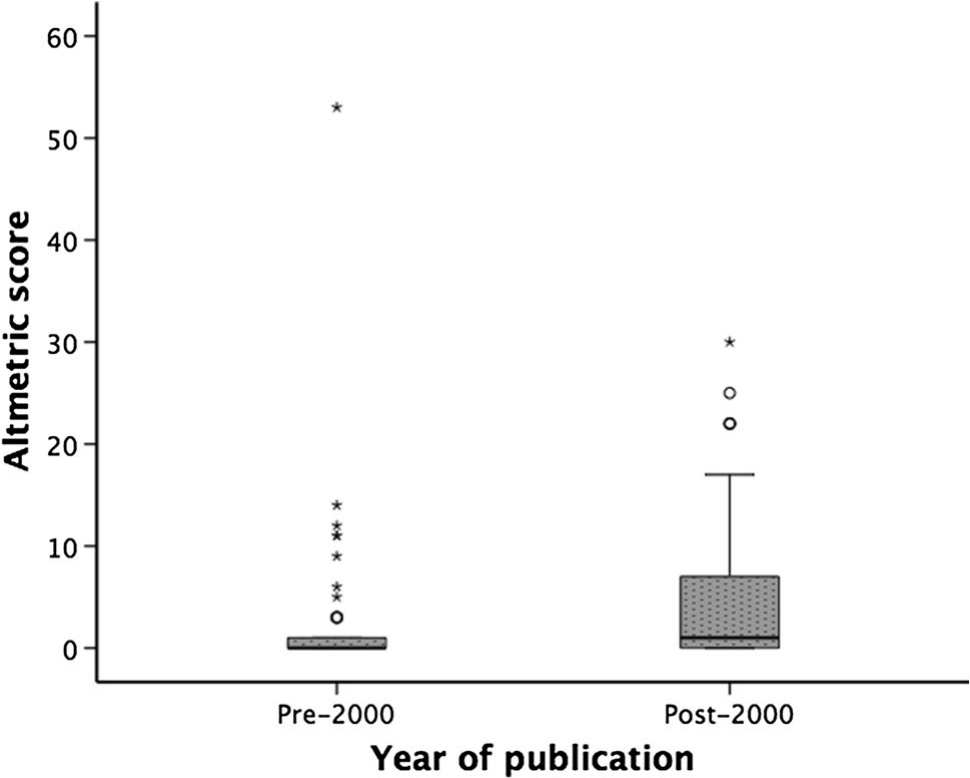
The distribution of Altmetric scores in articles published pre- and post-2000. *Kruskal–Wallis test *p* = 0.018

**Fig. 3:**
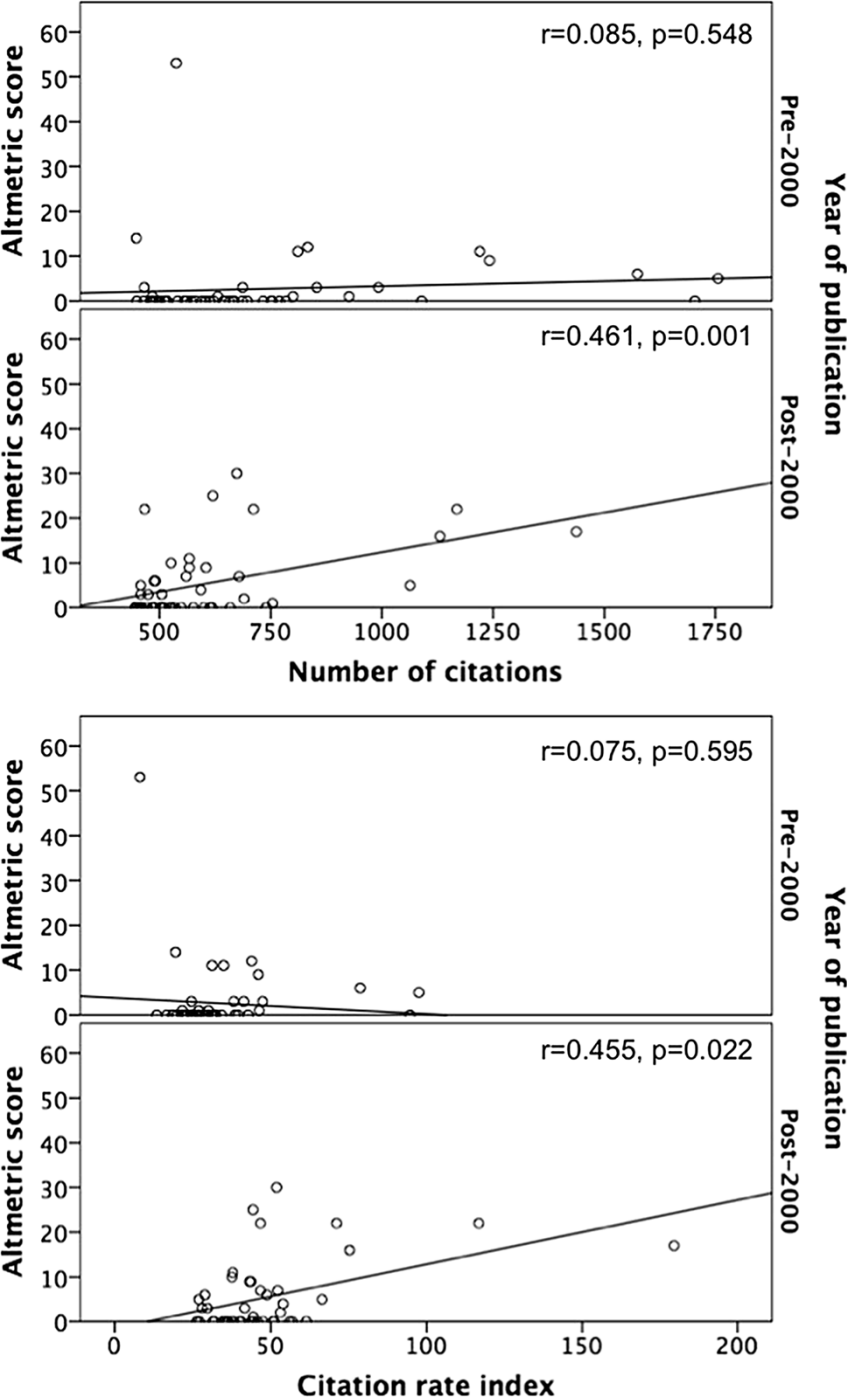
The relationship between Altmetric score, number of citations, citation rate index stratified by pre- and post-2000 publication

Altmetric scores ranged from 0 to 53 (median 0) with 40 articles scoring ≥ 1.0. The article with the highest AS was by Bigelow et al. [69] (Table 4). The USA had the most articles in the top 10 AS with 4, followed by Canada and Sweden with 2 each. Clinical guidelines (*n* = 3) were the commonest topic in the top 10 AS, followed by characterisation and management of complications (*n* = 2). Articles published from the year 2000 onwards had a significantly higher AS (*p* = 0.018) with a median of 1.0 (IQR 0.0–9.0), compared with a median of 0.0 (0.0–1.0) in articles published before 2000 (Fig. 2). AS correlated with citation rate index (*r* = 0.266, *p* = 0.008), but not with total number of citations (*r* = 0.179, *p* = 0.079). In articles published after 2000, AS was associated with number of citations (*r* = 0.461, *p* = 0.001), and citation rate index (*r* = 0.455, *p* = 0.002). This correlation was not evident in articles published before 2000 for number of citations (*r* = 0.085, *p* = 0.548) or for citation rate index (*r* = 0.075, *p* = 0.595, Fig. 3). Twenty-three articles appeared in both the top 40 for citations and AS. AS was not associated with evidence level when grouped as therapeutic/aetiology studies (SCC = 0.149, *p* = 0.244), prognostic studies (SCC − 0.277, *p* = 0.197), or journal impact factor (*r* = 0.160, *p* = 0.118).

**Table 4:** Top 10 articles with the highest Altmetric score

Rank	Altmetric score	First author	Senior author	Title	Institution	Country
1	53.0	WG, Bigelow	WF, Greenwood	Hypothermia—Its possible role in cardiac surgery—An investigation of factors governing survival in dogs at low body temperatures	University of Toronto	Canada
2	30.0	NV, Christou	LD, MacLean	Surgery decreases long-term mortality, morbidity, and health care use in morbidly obese patients.	McGill University	Canada
3	25.0	B, Brandstrup	F, Pott	Effects of intravenous fluid restriction on postoperative complications: comparison of two perioperative fluid regimens: a randomized assessor-blinded multicenter trial	H:S Bispebjerg University Hospital	Denmark
4	22.0	RM, Rosenfeld	DL, Witsell	Clinical practice guideline: adult sinusitis	SUNY Downstate Medical Center and Long Island College Hospital	USA
5	22.0	L, Norgren	FGR, Fowkes	Inter-society consensus for the management of peripheral arterial disease (TASC II)	Orebro University	Sweden
6	22.0	L, Norgren	CD, Liapis	Inter-society consensus for the management of peripheral arterial disease (TASC II)	Orebro University	Sweden
7	17.0	PA, Clavien	M, Makuuchi	The Clavien-Dindo Classification of Surgical Complications Five-Year Experience	University Hospital of Zurich	Switzerland
8	16.0	NE, Seymour	RM, Satva	Virtual reality training improves operating room performance—Results of a randomized, double-blinded study	Yale University School of Medicine	USA
9	14.0	EH, Oldfield	NJ, Patronas	Pathophysiology of syringomyelia associated with Chiari I malformation of the cerebellar tonsils—Implications for diagnosis and treatment	National Institute of Neurological Disorders and Stroke	USA
10	12.0	SF, Khuri	JF, Stemple	The Department of Veterans Affairs’ NSQIP: the first national, validated, outcome-based, risk-adjusted, and peer-controlled program for the measurement and enhancement of the quality of surgical care. National VA Surgical Quality Improvement Program	Brockton/West Roxbury VA Medical Center	USA

## Discussion

Periodical journals have been the principal means of disseminating scientific research since the seventeenth century, with the oldest still in existence, the *Philosophical Transactions of the Royal Society,* appearing first in 1665. Over the intervening three and a half centuries, journals have established conventions for publication; insisting on independent (and usually anonymous) peer review of submissions, intended to preserve the integrity of the scientific process, but have come under increasing recent scrutiny. This bibliometric and Altmetric analysis is the first of its kind to identify the authors and themes that have had the greatest impact within the global arena of surgery. Several different pathological conditions and surgical interventions were included within this diverse field, as demonstrated by the top 100 articles relating to some six different anatomical and physiological systems and subject areas. A surprising finding was that no correlation was found between citation number and level of evidence, as defined by objective evidence based medicine references, which may seem counterintuitive, yet likely represents the challenge of linking impact with citation and research quality. Altmetric scores represent the latest emerging development and, in a decade or less, have clearly had significant impact because AS was significantly associated with both citation number and citation rate.

Citation rates were higher for recently published articles, which imply that these will become more influential clinically within the next 5–10 years. Influential articles are more likely to be cited by the scientific community, and these citations form the basis of the impact factor; which quantifies the average citations of the articles published within the journal during a specific period. Journals with a higher impact factor are recognised as being of a higher quality and more likely to contain influential articles. The majority of articles were published in journals with an impact factor of less than 5.7. Annals of Surgery published the most articles ranked within the upper 100 (*n* = 32) and has historically been the surgical journal ranked highest with an impact factor of 8.7. Journals with high-impact factors (54.42–29.35), NEJM, Lancet, JAMA, and Nature Genetics, were not represented in this analysis, yet the citations accrued by the articles identified were similar to those reported in other bibliometric analyses; colorectal cancer (7850–989) [109], gastric cancer (2893–299) [4], and oesophageal cancer (1833–293) [110]. A possible explanation relates to the novelty of the results, which may be classified relating to the scientific community in general, or confined to the field studied. Findings already reported in other arenas may then be re-established in the arena of surgery, and such articles are unlikely to be published in high-impact scientific journals, yet within the context of this study, remain likely to be considered influential.

There are a number of potential limitations inherent within this study related to several types of bias, which may confound the results. Disproportionate multiple citations may result from institutional bias, self-citation, powerful person, or language bias. Geographically, the high rate of publications from the USA has been reported previously [4, 5], arguably reflecting a preference by USA institutions to cite research performed locally. This effect may have been amplified by limiting the search to English-language articles. Moreover, older articles have a greater citation potential simply because of the length of time they have been in the public domain, rather than representing a true measure of impact. In an attempt to control for this, the number of citations was divided by the number of years since publication: a citation rate index. Despite these measures, the lead-time for publication of citing articles may still result in under representation of more recent articles. The inclusion of only first and senior authors and the institution of the first author is also a possible limitation of the study, as it is possible that several first authors may have co-authored other articles, and are therefore under represented. The search terms used also represent an inherent weakness associated with interrogating bibliographic repositories, in that no combination of search terms is perfect. To identifying all papers relevant to surgery would arguably require an almost infinite number of search terms, which is not pragmatic. While the relative limitation of the search term ‘surgery’ is acknowledged, it is unlikely that this methodology has had a material effect on the ability of the study to address the a priori hypotheses. In contrast, the study has strengths. The many-landmark, randomized trials of surgical procedures, published in scientific and general medical journals, were included by the search methodology, which included the search term surgery, and encompassed non-surgical journals such as Nature, Science, NEJM, and the Lancet. The use of the search term surgery, more so than procedure or intervention, ensures that the results retain relevance to the field of surgery.

Another role that academic journals have come to play, and not part of their original job-description of disseminating scientific results, is as a marker of a researcher’s prowess, and thus a surrogate determinant of academic career potential. Publication in a blue-chip title such as *Nature* or *Science,* without question represents a feather in the cap of any clinician, and therefore unlikely to be overlooked by any academically focused appointment committee. An article’s true quality is better revealed by the number of times it is cited elsewhere (ideally not self-citation), but citations take time to accumulate, and other, faster assessment means would be welcome, which has inevitably led to the development of alternative metrics in this regard, now termed “altimetry”. These extend the concept of citation beyond references in other scientific papers; by recording, for example, how often a paper is downloaded, or when the outcome of a clinical trial is used to develop guidelines for doctors, or if a piece of work is included in a course curriculum. Altmetric.com, based in London, was one of the first companies to work in this area, and since 2011, it has tracked mentions of published papers in sources ranging from social media and Wikipedia, to policy documents published by government departments. A rival firm, Plum Analytics, in Philadelphia, tracks mentions, downloads, clicks and the like, of everything from preprints (papers that have been made publicly available, but are not yet formally published), and sets of raw data, to non-commercial computer programs which investigators have written to assist their own endeavours. Using Altmetrics should therefore indicate the importance of a wider range of research-related activities than citations provide, and moreover, do so faster. Plum Analytics was bought by Elsevier in 2017, one of the world’s largest scientific publishers; suggesting that Altmetrics may also prove profitable, as well as useful.

Findings of medical research have long been considered to be disseminated too slowly, but that is about to change. In January 2017 the Gates Foundation introduced a policy, that research it supports (it is the world’s biggest source of charitable money for scientific endeavours, to the tune of some $4bn a year) must, when published, be available to all, and followed this by announcing that it will foot the cost of placing such research in one repository of freely available articles, meaning that Gates-sponsored research cannot be invoiced. Such a manoeuvre would carry the caveat that recipients of Gates’ financial support can no longer offer their output to journals such as *Nature*, or the *New England Journal of Medicine,* since accessing the content of these publications has associated cost. Their prestige is based on their ability to pick and publish only the best. If some work is out of bounds to these journals, no matter how good, that will risk diminishing their quality, and arguably those journals’ businesses could suffer and even crumble. Moreover, by actively directing the beneficiaries of its patronage towards the repository in question, set up last year by the Wellcome Trust (after Gates, the world’s second-largest medical research charity), the foundation is pointing to a specific type of alternative, and a future scientific publication arena that, if not completely journal-free, is likely journal-light.

## Conclusion

This list of the top-cited articles in surgery has worth for a number of reasons. It identifies seminal contributions, facilitates the understanding and development of contemporary surgical history; and offers clues regarding what makes an article a likely top-cited classic. To produce such a work, the author or research group must come up with a clinical or nonclinical innovation, observation, or discovery that has a potential long-standing effect on surgical clinical practice. Based on the findings of this study, to be rewarded by citation number, such a scholarly contribution should be published in a high-impact journal, is more likely to be amplified and resonate loudly if it originates from an Anglo-Saxon academic institute, and in view of the dissonance regarding level of evidence, needs to be noticed by surgeons active on social media, implying a deal of good fortune is required. Recent studies have identified Twitter as the most commonly used smartphone application by surgeons, but the rates of engagement are variable between medical specialties; for example colorectal surgeons’ engagement levels lag behind other disciplines. In 2014, only 31% of UK consultant colorectal surgeons were found to have Twitter profiles, compared with higher engagement rates within other surgical sub-specialties such as urology (33%), plastic surgery (22%) and vascular surgery (5%) [111]. To make any best seller list has now become that much more challenging.

## Electronic supplementary material

Below is the link to the electronic supplementary material.

Supplementary material 1 (DOCX 133 kb)
