# Supplementary material for: Altmetric Versus Bibliometric Perspective Regarding Publication Impact and Force
Source: World J Surg. 2018 Mar 13;42(9):2745–56. doi: 10.1007/s00268-018-4579-9 (PMC6097753; doi:10.1007/s00268-018-4579-9)
Supplement: Supplementary file 1 — Supplementary material 1 (DOCX 133 kb) [file 268_2018_4579_MOESM1_ESM.docx]

Supplementary table 1. Institutions with the highest number of papers in the top 100

| **Institution** | **Number of articles in top 100** | **Total number of citations** |
| --- | --- | --- |
| Memorial Sloan-Kettering Cancer | 4 | 3307 |
| Washington University | 4 | 2434 |
| University of Zurich | 3 | 7644 |
| University of Texas | 3 | 2356 |
| University of Toronto | 3 | 2065 |
| John Hopkins University | 3 | 1400 |
| Orebro University | 2 | 1879 |
| University of Virginia | 2 | 1486 |
| Hospital Paul Brousse | 2 | 1371 |
| University of Pittsburgh | 2 | 1359 |
| University of California Los Angeles | 2 | 1222 |
| Baylor College of Medicine | 2 | 1202 |
| Cleveland Clinic Foundation | 2 | 1170 |

Supplementery table 2. General issues

| **Specialty** | **Number of articles** |
| --- | --- |
| HPB | 15 |
| Trauma & Orthopaedic | 13 |
| Cardiothoracic | 11 |
| Colorectal | 10 |
| Neurosurgery | 10 |
| Vascular | 8 |
| Bariatric | 6 |
| General Surgery | 6 |
| ENT | 4 |
| Multiple | 5 |
| Breast | 2 |
| Teaching | 2 |
| Ophthalmology | 1 |
| Plastics | 1 |
| Urology | 1 |

HPB, Hepatico-pancreato-biliary; ENT, Ear Nose and Throat.

Supplementery table 3. Specific fields

| **Subject** | **Number of articles** |
| --- | --- |
| Management  Surgical treatment  Randomised Control Trial  Surgical technique | 53  46  14  7 |
| Surgical Complications | 16 |
| Pathology | 8 |
| Prognosis | 5 |
| Training Methods | 5 |
| Guidelines | 4 |
| Epidemiology | 3 |
| Quality of Life | 3 |
| Reporting Methods | 2 |
| Nutrition | 1 |

**Supplementary figure 1.** The distribution of the number of citations between studies relating to clinical trials in surgery


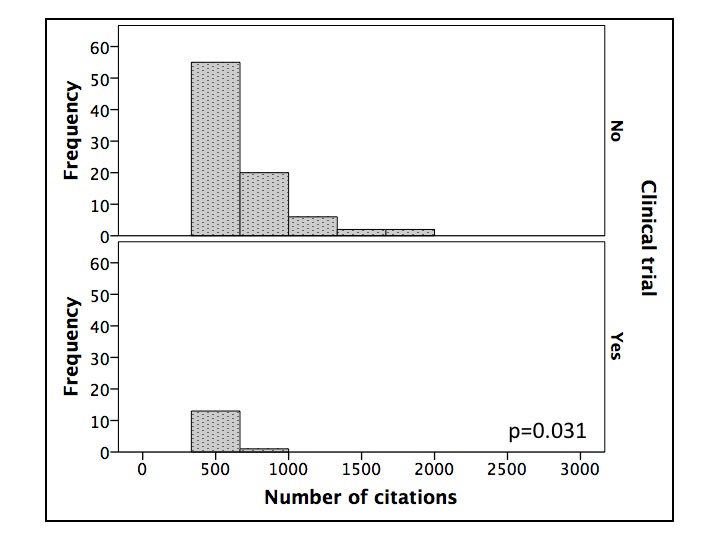


* The highest cited manuscript by Dindo et al was an outlier with a citation of 6336. This was removed for graphical representation to make the distribution of citations clearer.

Supplementery figure 2. The relationship between number of citations and Sacket score


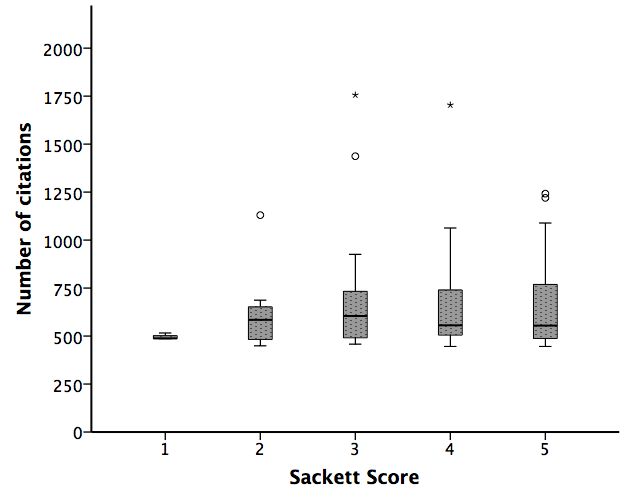


* p=0.674, Kruskal-Wallis Test
